# Prevalence and risk factors of sarcopenia and effect of sarcopenia on functional status and falls incidents among the elderly in Selangor

**DOI:** 10.7717/peerj.20175

**Published:** 2025-10-17

**Authors:** Thinakaran Kandayah, Nazarudin Safian, Shamsul Azhar Shah

**Affiliations:** Department of Public Health Medicine, Faculty of Medicine, Universiti Kebangsaan Malaysia, Cheras, Kuala Lumpur, Malaysia

**Keywords:** Prevalence, Risk factors, Functional status, Falls, Sarcopenia, Elderly

## Abstract

**Background:**

The burden of sarcopenia is increasing but studies on sarcopenia at the population level are limited in Malaysia. This study was conducted to identify the prevalence, risk factors and effect of sarcopenia on functional status and falls among the elderly in Selangor state.

**Methods:**

Anthropometry, body composition measurements and face-to-face interview using questionnaires on functional status and falls were conducted on 469 respondents. Prior to the interview, written informed consent was obtained from the respondents. The inclusion criteria for this study is being 60-years old and above, able to understand, read and speak Bahasa Malaysia or English language and voluntarily consents to participates in the study. Multifrequency bioelectrical impedance analysis (BIA) was used to measure the body composition. Sarcopenia assessment was done using the guideline from Asian Working Group for Sarcopenia (AWGS) 2019.

**Results:**

The prevalence of possible sarcopenia, confirmed sarcopenia and severe sarcopenia were 38.4%, 10.0% and 24.5%, respectively. Prevalence of activities of daily living (ADL) and instrumental activities of daily living (IADL) dependence were 26.0% and 25.4%, respectively and 42.2% of the respondents experienced falls in the last 12 months. Multinomial logistic regression model showed that locality (AOR = 2.90; *p* < 0.001), type-2 diabetes mellitus (adjusted odds ratio (AOR) = 1.87; *p* = 0.031) and female gender (AOR = 2.58; *p* < 0.001) were significantly associated with possible sarcopenia. Female gender (AOR = 3.04; *p* = 0.005) and depression (AOR = 3.27; *p* = 0.048) were significantly associated with confirmed sarcopenia, where else hypertension (AOR = 0.45; *p* = 0.039) were found to be a protective factor for confirmed sarcopenia. Age (AOR = 4.52; *p* < 0.001), female gender (AOR = 1.84; *p* = 0.045), race (AOR = 3.82; *p* = 0.001), locality (AOR = 3.82; *p* < 0.001), level of education (AOR = 5.32; *p* = 0.010) and physical activity (AOR = 2.28; *p* = 0.029) were significantly associated with severe sarcopenia. The respondents with confirmed sarcopenia and severe sarcopenia were significantly associated with ADL (AOR = 10.54; *p* < 0.001) and IADL (AOR = 8.55; *p* < 0.001) dependence after adjustment for the covariates. In addition, after adjusting for covariates, respondents with possible sarcopenia (AOR = 3.34; *p* < 0.001) and respondents with confirmed sarcopenia and severe sarcopenia (AOR = 10.62; *p* < 0.001) were significantly associated with falls incidents.

**Conclusions:**

The findings from this study highlights the detrimental effects of sarcopenia and the importance of early detection at the community level.

## Introduction

Sarcopenia is a chronic disease characterized by the loss of muscle mass, muscle strength and function in the elderly (Tarantino et al., 2023; Xie et al., 2021). The term sarcopenia was introduced by Rosenberg, who discussed the concept of age related decrease in muscle mass (Rosenberg, 1989; Rosenberg, 1997). Subsequently, European Working Group on Sarcopenia in Older People (EWGSOP) was developed in 2010 and followed by Asian Working Group for Sarcopenia (AWGS) in 2014 (Cruz-Jentoft, 2010; Chen et al., 2014). The aforementioned guidelines were then updated in 2019 (Cruz-Jentoft et al., 2019; Chen et al., 2020). In order to develop a universally accepted and standardized definition of sarcopenia, Global Leadership Initiative in Sarcopenia (GLIS) was initiated and a steering committee was formed in 2019–2021. Agreement were reached through Delphi study on general aspect, components and outcome of sarcopenia (Kirk et al., 2024). The aforementioned refined guideline provided a clearer diagnostic algorithm for the researchers, intensifying studies on sarcopenia globally.

The pathophysiology of sarcopenia is related to factors such as imbalance between the catabolic and anabolic pathway of muscle protein, reduction in satellite cells and neuromuscular junction dysfunction (Jang, Kim & Kim, 2023; Patel et al., 2015; Moreira-Pais et al., 2022). There are various complications associated with sarcopenia which are concerning. Studies have shown that, elderly people suffering from sarcopenia are associated with higher mortality, falls, fracture, hospitalization rates and functional status dependence compared to those without sarcopenia (Beaudart et al., 2016; Hunter et al., 2019; Yeung et al., 2019; Wearing et al., 2020).

The prevalence of sarcopenia globally is around 10.0% to 16.0% based on a study in Sweden (Yuan & Larsson, 2023). However, the prevalence of sarcopenia at the Asian region differs, ranging from 5.5% to 25.7%, with the incidence of sarcopenia being more prevalent in men (5.1–21.0%) than in women (4.1–16.3%) (Chen et al., 2020). Meanwhile in Malaysia, the prevalence of sarcopenia ranges between 3.6% to 28.5% (Ramoo et al., 2022; Sazlina et al., 2020).

According to the latest guidelines of AWGS 2019, updates were done on the definition of low muscle strength and low physical performance. Besides that the term possible sarcopenia were introduced with the purpose of early intervention (Chen et al., 2020). At present, studies with the updated guideline are limited in Malaysia (Ranee et al., 2022; Mohamad Zani et al., 2025). In addition, the AWGS 2019 also recommends using bioelectrical impedance analysis (BIA) with multiple frequency instead of single frequency, as it may compromise the diagnosis. Besides that, most of the studies pertaining to sarcopenia in Malaysia still uses BIA with single frequency, hence this study was justified as it used BIA with multiple frequency (Ramoo et al., 2022; Mohamad Zani et al., 2025).

Clinical practice guidelines specifically for sarcopenia are unavailable at the moment in Malaysia. Clinical practice guidelines are important as it helps doctors to improve the outcome of patient health care (Guerra-Farfan et al., 2023). Hence this study output could be used as one the key data when creating the clinical practice guidelines in Malaysia, especially in terms of disease burden and risk factors of sarcopenia.

In addition, previous sarcopenia studies in Malaysia were more focused on the elderly population suffering from specific diseases such as diabetes mellitus and cognitive impairment (Ramoo et al., 2022; Sazlina et al., 2020). However, comprehensive research at the community level on sarcopenia disease burden, risk factors and the effects are limited. Hence, we conducted this research at the community level to investigate the prevalence, risk factors and the effect of sarcopenia on functional status and falls among elderly in Selangor state, Malaysia.

## Materials and Methods

### Design and the study population

This is a cross-sectional study which was conducted among elderly aged 60 years and above in two districts in Selangor. The state of Selangor was chosen as the study location because it is one of the state with a high elderly population in Malaysia (DOSM, 2024a). In addition, Selangor state is the highest populated state in Malaysia making up 21.6 percent of the nation’s population and comprises of all major races such as Malay, Chinese and Indian (DOSM, 2024b). As we aimed to investigate the disease burden and effects of sarcopenia, a diverse elderly population is essential to ensure generalizable study outcome. Kuala Langat district with an estimated population of 0.28 million and Petaling district with an estimated population of 2.2 million was chosen to represent the rural and urban region respectively (DOSM, 2021). Sampling frame of this study was derived from the Community Development Unit under the district office. Subsequently, the list of elderly people from the areas selected was obtained after engagement with the village or community head. Multistage cluster sampling was performed with Kuala Langat and Petaling district as the primary sampling units. Three sub-districts from each district was chosen as the secondary sampling unit. Finally, a town or village were randomly chosen from each sub-district as the third sampling point. Random sampling was done for households with elderly above 60 years old. Whenever more than one adult is present in the chosen household, Kish grid table was used to pick the sample. This method helps to eliminate any potential bias by the researcher from selecting respondents themselves, and ensuring each member of the household who meets the eligibility has an equal chance of being chosen (Nemeth, 2002). The inclusion criteria for this study is being 60-years old and above, able to understand, read and speak Bahasa Malaysia or English language and voluntarily consents to participates in the study.

The sample size of this study was determined using the Kish formula, together with design effect of 1.2. With the consideration of 20% of non response rate, the sample for this study was 451. We were able to recruit 494 respondents and after excluding 25 respondents who did not fulfil the inclusion criteria, 469 respondents were accepted to participate in the study voluntarily without any conflict of interest. The age range of the respondents who participated in this study were from 60 years old to 88 years old of age.

Prior to the interview, respondents were briefed regarding the study and given the information sheet and consent form. Subsequently, written informed consent from the participants was obtained and the interview was conducted face to face by the researchers and used either Bahasa Malaysia or English language. Each interview session lasted about 30–40 min. In this study, apart from the interview session, anthropometry and body composition measurements were performed on the respondents.

This study received approval from the Research Ethics Committee of University Kebangsaan Malaysia, under the approval code FF-2022-363. This research was conducted according to the the principles of the Declaration of Helsinki.

### Study variables

The respondents of this study answered the questionnaires regarding sociodemographic, socioeconomic status, lifestyle and presence of comorbidities. Physical activity level was determined using the Global Physical Activity Questionnaire (GPAQ) (WHO, 2002). Depression were determined using the Geriatric Depression Scale (GDS) (Yesavage et al., 1982). Barthel Index of Activities of Daily Living and Lawton-Brody Instrumental Activities of Daily Living questionnaires were used to access functional status (Mahoney & Barthel, 1965; Lawton & Brody, 1969). Falls incidents were determined using the 12-months falls recall questionnaire (NIH, 2018a). Questionnaires for the functional status and falls incidents were translated to Bahasa Malaysia and used in National Health and Morbidity Survey (NHMS) 2018 elderly health survey (NIH, 2018a). Prior to the study, necessary permission to use the translated and validated questionnaires into Bahasa Malaysia were obtained. Cultural adaptation on the questionnaires were not performed in this study due to cost and time constraints.

### Diagnosis of sarcopenia

In this study, possible sarcopenia, confirmed sarcopenia and severe sarcopenia were determined using the AWGS 2019 criteria (Chen et al., 2020). Possible sarcopenia is determined by low muscle strength or reduced physical performance. Low muscle mass and poor muscle strength or physical performance indicates confirmed sarcopenia. Presence of low muscle mass, muscle strength together with reduced physical performance indicates diagnosis of severe sarcopenia. Using BIA, the cut-offs for muscle mass measurement are <7.0 kg/m^2^ for men and <5.7 kg/m^2^ for women. Low muscle strength is defined as handgrip strength <28 kg for men and <18 kg for women. Low physical performance is indicated with 5-time chair stand test that took 12 s or more to be completed.

Height of the respondents were measured in meter using the Inbody PUSH, a portable ultrasonic digital stadiometer (Inbody, 2016). Before the measurement was taken, the procedure was explained and the respondent was asked not to move during the measurement. Next, the researcher ensured that the respondent had removed their shoes or slippers and was asked to stand on the floor with even surface. The end of the portable stadiometer were placed on the head of the respondent and the measurement was taken once and recorded. If the respondent moved during the measurement, the process of measurement was repeated again.

Appendicular skeletal mass of the respondents were accessed using the portable multifrequency bioimpedance analysis device (InBody 270; Seoul, Korea). Muscle strength was determined using the hand grip dynamometer (T.K.K. 5001 GRIP-A; Takei Scientific Instrument Co. Ltd., Japan). The hand grip strength of each respondents was measured three times in standing position by alternating both hands and the highest average value were chosen. Physical performance were performed using the 5-time chair stand test and the time taken were recorded using stopwatch. Respondents that took 12 s or more were classified as low physical performance (Chen et al., 2020).

### Data analysis

Complete data were obtained and analysed using IBM SPSS version 21.0 (IBM Corp., Armonk, NY, USA) with the significance level of *p* < 0.05 (two-tailed). Descriptive data with categorical variables were analysed using frequency and percentage. In order to address the effect of confounders, multivariate analysis was used. Multinomial logistic regression that comprises of univariate and multivariate analysis was used to examine the relationship between sociodemographic factors, socioeconomic factors, lifestyle, and comorbidities with sarcopenia. The statistical test requirements were met and all variables were checked for interaction. No significant interaction was found between the variables. In addition, multi-collinearity between variables was tested and the variance inflation factor (VIF) value was less than ten and the standard error value was less than five. Based on the Model Fitting Information, the Chi-Square test produced a value of (*p* < 0.05), which indicated that there was a significant relationship between the dependent and independent variables in the final model. The goodness of fit test with Pearson and Deviance statistical values (*p* > 0.05) showed that the model had a good fit with the data. The pseudo-R-Square values including Cox and Snell, Nagelkerke and McFadden showed that the variation explained by the model ranged from 12.6% to 30.2%

Binary logistic regression analysis which includes simple and multiple binary logistic were used to assess the effect of sarcopenia on functional status dependence and falls incidents among the elderly. Both backward and forward likelihood ratio (LR) tests produced similar and consistent results. Besides that, none of the variables had significant interactions. Multi-collinearity were determined with the value of VIF less than ten and the standard error value was less than five. The model used was fit as evidenced by the Hosmer-Lemeshow goodness-of-fit test which showed non-significant results. Besides that, adjustment for covariates were also performed in assessing the effect of sarcopenia on functional status dependence and falls incidents. In addition, bivariate analysis were used to analyse falls characteristics based on the sarcopenia classification.

## Results

Out of a total of 469 respondents, 226 (48.2%) were male respondents, whereas 243 (51.8%) were female respondents. A total of 354 (75.5%) respondents in this study were in the age category of 60 to 74 years. Besides that, majority of respondents were Malays (38.0%), followed by Indian respondents (34.1%) and Chinese respondents (27.9%). In this study, 52.0% of the respondents have primary education level or less and 88.3% of the respondents were married.

The prevalence of possible sarcopenia, confirmed sarcopenia and severe sarcopenia was 38.4%, 10.0% and 24.5%, respectively. The prevalence of activities of daily living (ADL) and instrumental activities of daily living (IADL) dependency was 26.0% and 25.4% respectively and 42.2% of the respondents reported falls in the past 12 months. Other descriptive statistics were shown in Table 1. Meanwhile, risk factors associated with sarcopenia were shown in Table 2.

**Table 1:** Characteristics of the study population.

Variables		(*n*)	(%)
Age (years)	60–74	354	75.5
	≥75	115	25.5
Gender	Male	226	48.2
	Female	243	51.8
Race	Malay	178	38.0
	Chinese	131	27.9
	Indian	160	34.1
Education level	Primary or less	244	52.0
	Secondary	184	39.2
	Tertiary	41	8.7
Marital status	Single	15	3.2
	Married	414	88.3
	Widowed or divorced	40	8.5
Locality	Petaling	234	49.9
	Kuala Langat	235	50.1
Occupation	Not employed	94	20.0
	Housewife	212	45.2
	Retired	70	14.9
	Working	93	19.8
Household income	B-40	452	96.4
	M-40	11	2.3
	T-20	6	1.3
Smoking status	Active smoker	50	10.7
	Ex-smoker	37	7.9
	Never smoked	382	81.4
Alcohol consumption status	Alcohol drinker	98	20.9
	Ex-drinker	9	1.9
	Never drinked	362	77.2
Physical activity	Active	109	23.2
	Inactive	360	76.8
Type-2 diabetes mellitus	Yes	160	34.1
	No	309	65.9
Hypertension	Yes	298	63.5
	No	171	36.5
Dyslipidemia	Yes	294	62.7
	No	175	37.3
Cardiovascular disease	Yes	50	10.7
	No	419	89.3
Osteoporosis	Yes	29	6.2
	No	440	93.8
Depresssion	Yes	38	8.1
	No	431	91.9
Sarcopenia	No sarcopenia	127	27.1
Classification	Possible sarcopenia	180	38.4
	Confirmed sarcopenia	47	10.0
	Severe sarcopenia	115	24.5
ADL	Dependency	122	26.0
	No dependency	347	74.0
IADL	Dependency	119	25.4
	No dependency	350	74.6
Falls incidents	Yes	198	42.2
	No	271	57.8

**Table 2:** Multinomial multivariate logistic regression analysis for risk factors of sarcopenia.

Variables	Possible sarcopenia			Confirmed sarcopenia			Severe sarcopenia		
	β	SE	aOR (95% CI)	β	SE	aOR (95% CI)	Β	SE	aOR (95% CI)
**Age (years)**									
60–74	1.00								
≥75	0.533	0.332	1.71 [0.89–3.27]	0.663	0.470	1.94 [0.77–4.87]	1.509	0.353	4.52 [2.26–9.03]***
**Gender**									
Male	1.00								
Female	0.948	0.267	2.58 [1.53–4.36]***	1.112	0.392	3.04 [1.41–6.55]**	0.610	0.304	1.84 [1.01–3.34]*
**Race**									
Malay	0.499	0.319	1.65 [0.88–3.08]	−0.207	0.447	0.81 [0.34–1.95]	0.327	0.381	1.39 [0.66–2.93]
Indian	0.509	0.362	1.66 [0.82–3.38]	0.400	0.493	1.49 [0.57–3.92]	1.339	0.401	3.82 [1.74–8.39]**
Chinese	1.00								
**Locality**									
Petaling	1.066	0.260	2.90 [1.75–4.83]***	0.014	0.395	1.01 [0.47–2.20]	1.339	0.304	3.82 [2.10–6.93]***
Kuala Langat	1.00								
**Level of education**									
Primary or less	0.368	0.440	1.45 [0.61–3.42]	1.006	0.850	2.73 [0.52–14.47]	1.671	0.649	5.32 [1.49–18.97]*
Secondary	−0.157	0.427	0.85 [0.37–1.98]	0.850	0.832	2.34 [0.46–11.96]	0.572	0.645	1.77 [0.50–6.28]
Tertiary	1.00								
**Physical activity**									
Active	1.00								
Inactive	0.403	0.300	1.50 [0.83–2.69]	−0.149	0.402	0.86 [0.39–1.89]	0.825	0. 377	2.28 [1.09–4.78]*
**Comorbidities**									
Type-2 diabetes mellitus									
Yes	0.627	0.290	1.87 [1.06–3.31]*	−0.291	0.486	0.75 [0.29–1.94]	0.545	0.335	1.73 [0.89–3.33]
No	1.00								
**Hypertension**									
Yes	0.199	0.281	1.22 [0.70–2.12]	−0.811	0.393	0.45 [0.21–0.96]*	−0.592	0.321	0.55 [0.30–1.04]
No	1.00								
**Depression**									
Yes	−0.594	0.543	0.55 [0.19–1.60]	1.183	0.600	3.27 [1.01–10.59]*	−0.103	0.552	0.90 [0.31–2.66]
No	1.00								

**Notes:**

1.00 = reference category, SE = standard error, β = Beta, Nagelkerke pseudo-R2 = 0.30.

* *p* < 0.05.

** *p* < 0.01.

*** *p* < 0.001.

### Possible sarcopenia

#### Risk factors

Female respondents had 2.6 times higher odds (adjusted odds ratio 2.58, 95% confidence interval (CI) [1.53–4.36], *p* < 0.001) of having possible sarcopenia than no sarcopenia compared to male respondents. Based on the locality, respondents who live in the Petaling district have 2.9 times higher odds (adjusted odds ratio 2.90, 95% CI [1.75–4.83], *p* < 0.001) to have possible sarcopenia than no sarcopenia compared to respondents who live in the Kuala Langat district. In addition, respondents with type-2 diabetes mellitus had 1.9 times higher odds (adjusted odds ratio 1.87, 95% CI [1.06–3.31], *p* = 0.031) to have possible sarcopenia than no sarcopenia compared to respondents who do not have type-2 diabetes mellitus.

### Confirmed sarcopenia

#### Risk factors

Female respondents had 3 times higher odds (adjusted odds ratio 3.04, 95% CI [1.41–6.55], *p* = 0.005) of having confirmed sarcopenia than no sarcopenia compared to male respondents. Respondents with depression had 3.3 times higher odds (adjusted odds ratio 3.27, 95% CI [1.01–10.59], *p* = 0.048) of having confirmed sarcopenia than no sarcopenia compared to respondents without depression. Respondents with hypertension had 0.45 times lower odds (adjusted odds ratio 0.45, 95% CI [0.21–0.96], *p* = 0.039) of having confirmed sarcopenia than no sarcopenia compared to respondents without hypertension.

### Severe sarcopenia

#### Risk factors

Respondents in the age category 75 years and above had 4.5 times higher odds (adjusted odds ratio 4.52, 95% CI [2.26–9.03], *p* < 0.001) of having severe sarcopenia than no sarcopenia compared to respondents in the age category 60 to 74 years. Female respondents had 1.8 times higher odds (adjusted odds ratio 1.84, 95% CI [1.01–3.34], *p* = 0.045) of having severe sarcopenia than no sarcopenia compared to male respondents. Respondents living in Petaling district had 3.8 times higher odds (adjusted odds ratio 3.82, 95% CI [2.10–6.93], *p* < 0.001) of having severe sarcopenia than no sarcopenia compared to respondents living in Kuala Langat district.

In this study it was found that Indian respondents had 3.8 times higher odds (adjusted odds ratio 3.82, 95% CI [1.74–8.39], *p* < 0.001) of having severe sarcopenia than no sarcopenia compared to Chinese respondents. Based on the level of education, respondents with primary education or less were found to have 5.3 times more odds higher (adjusted odds ratio 5.32, 95% CI [1.49–18.97], *p* = 0.010) to have severe sarcopenia than no sarcopenia compared to respondents with tertiary education. Additionally, physically inactive respondents had 2.3 times higher odds (adjusted odds ratio 2.28, 95% CI [1.09–4.78], *p* = 0.029) of having severe sarcopenia than no sarcopenia compared to physically active respondents.

### Effect of sarcopenia on ADL dependency

Effect of sarcopenia on ADL dependency were shown in Table 3. After adjusting for age and osteoporosis, respondents with confirmed sarcopenia and severe sarcopenia had 10.5 times higher odds (adjusted odds ratio 10.54, 95% CI [4.86–22.85], *p* < 0.001) to have ADL dependency compared to respondents without sarcopenia.

**Table 3:** Multiple binary logistic regression analysis on effect of sarcopenia on ADL dependency.

Variables	β	SE	OR (95% CI)	β	SE	aOR (95% CI)
**Sarcopenia classification**						
No sarcopenia	1.00			1.00		
Possible sarcopenia	0.664	0.377	1.94 [0.93–4.06]	0.558	0.412	1.75 [0.78–3.92]
Confirmed sarcopenia and severe sarcopenia	2.405	0.352	11.08 [5.56–22.10]***	2.355	0.395	10.54 [4.86–22.85]***

**Notes:**

Adjusted for age and osteoporosis.

*** *p*-value < 0.001.

### Effect of sarcopenia on IADL dependency

Effect of sarcopenia on IADL dependency were shown in Table 4. After adjusting for age, occupation, high blood pressure, osteoporosis and depression, it was found that respondents with confirmed sarcopenia and severe sarcopenia had 8.5 times higher odds (adjusted odds ratio 8.55, 95% CI [3.76–19.44], *p* < 0.001) to have IADL dependency compared to respondents without sarcopenia.

**Table 4:** Multiple binary logistic regression analysis on effect of sarcopenia on IADL dependency.

Variables	β	SE	OR (95% CI)	β	SE	aOR (95% CI)
**Sarcopenia classification**						
No sarcopenia	1.00					
Possible sarcopenia	1.116	0.395	3.05 [1.41–6.62]**	0.789	0.435	2.20 [0.94–5.17]
Confirmed sarcopenia and severe sarcopenia	2.450	0.380	11.59 [5.50–24.40]***	2.146	0.419	8.55 [3.76–19.44]***

**Notes:**

Adjusted for age, occupation, Hypertension, osteoporosis and depression.

** *p*-value < 0.01.

*** *p*-value < 0.001.

### Effect of sarcopenia on falls incidents

Effect of sarcopenia on falls incidents were shown in Table 5. After adjusting for locality, physical activity and osteoporosis, respondents with the possible sarcopenia had 3.3 times higher odds (adjusted odds ratio 3.34, 95% CI [1.83–6.10], *p* < 0.001) to experience falls incidents compared to respondents with no sarcopenia. In addition, respondents with confirmed sarcopenia and severe sarcopenia had 10.6 times higher odds (adjusted odds ratio 10.62, 95% CI [5.77–19.52], *p* < 0.001) of experiencing a falls incidents compared to respondents with no sarcopenia.

**Table 5:** Multiple binary logistic regression analysis on effect of sarcopenia on falls incidents.

Variables	β	SE	OR (95% CI)	β	SE	aOR (95% CI)
**Sarcopenia classification**						
No sarcopenia	1.00					
Possible sarcopenia	1.396	0.296	4.04 [2.26–7.22]***	1.207	0.306	3.34 [1.83–6.10]***
Confirmed sarcopenia and severe sarcopenia	2.494	0.304	12.11 [6.67–21.98]***	2.362	0.311	10.62 [5.77–19.52]***

**Notes:**

Adjusted for locality, physical activity and osteoporosis.

*** *p*-value < 0.001.

### Falls characteristics based on sarcopenia classification

Falls characteristics based on sarcopenia classification were shown in Table 6. Out of the total 198 falls incidents, 90.9% of the falls occurred for respondents with possible sarcopenia, confirmed sarcopenia and severe sarcopenia compared to only 9.1% falls incidents that occurred for respondents with no sarcopenia.

**Table 6:** Bivariate analysis on characteristics of falls based on sarcopenia classification.

Falls characteristics	Total (*n*)	No sarcopenia *n* (%)	Possible sarcopenia *n* (%)	Confirmed sarcopenia and severe sarcopenia *n* (%)	$\chi$
**Falls incidents**					
Yes	198	18 (9.1)	72 (36.4)	108 (54.5)	81.01***
No	271	109 (40.2)	108 (39.9)	54 (19.9)	
**Frequency of falls**					
1	156	14 (9.0)	53 (34.0)	89 (57.0)	2.01
≥2	42	4 (9.5)	19 (45.2)	19 (45.2)	
**Type of injuries**					
Minor injury	179	13 (7.3)	63 (35.2)	103 (57.5)	10.63**
Severe injury	19	5 (26.3)	9 (47.4)	5 (26.3)	
**Treatment**					
Self treated	119	8 (6.7)	45 (37.8)	66 (55.5)	88.82***
Outpatient	61	5 (8.2)	19 (31.1)	37 (60.7)	
Admitted to hospital	18	5 (27.8)	8 (44.4)	5 (27.8)	
**Location of falls**					
Inside the house	104	6 (5.7)	37 (35.6)	61 (58.7)	100.95***
Bathroom/toilet	35	2 (5.7)	7 (20.0)	26 (74.3)	
House yard	39	7 (17.9)	21 (53.8)	11 (28.2)	
Outside of the house	20	3 (15.0)	7 (35.0)	10 (50.0)	

**Notes:**

$\chi$ = Chi square.

** *p*-value < 0.01.

*** *p*-value < 0.001.

According to the falls frequency, 42 participants in this research reported experiencing at least two falls, and 90.4% of them were classified as having possible sarcopenia, confirmed sarcopenia and severe sarcopenia, while only 9.5% of the respondents were with no sarcopenia.

We reported, 179 respondents had minor injuries and 92.7% of them occurred in respondents with possible sarcopenia, confirmed sarcopenia and severe sarcopenia compared to only 7.3% of respondents with no sarcopenia. Out of the total 19 incidents of severe injuries, 73.7% of them were suffered by respondents with possible sarcopenia, confirmed sarcopenia and severe sarcopenia compared to only 26.3% respondents with no sarcopenia. Out of the total 18 respondents who were admitted to the ward for falls related injuries, 72.2% of them were from possible sarcopenia, confirmed sarcopenia and severe sarcopenia, compared to only 27.8% of respondents with no sarcopenia.

According to the falls location, 104 falls incidents happened inside the house, and 94.3% of the respondents were having possible sarcopenia, confirmed sarcopenia and severe sarcopenia compared to only 5.7% of respondents with no sarcopenia. In total, 20 falls incidents occurred outside the house, and 85.0% of the respondents were having possible sarcopenia, confirmed sarcopenia and severe sarcopenia compared to only 15.0% of respondents with no sarcopenia.

## Discussion

### Prevalence of sarcopenia, functional status dependency and falls

We reported higher prevalence of possible sarcopenia, confirmed sarcopenia and severe sarcopenia compared to previous studies in Malaysia (Sazlina et al., 2020; Iskandar et al., 2021). However, this differences were not observed upon comparison with Southeast Asian and European countries, which reported higher prevalence on either possible sarcopenia, confirmed sarcopenia or severe sarcopenia (Pang et al., 2021; Murph et al., 2023; Vanitcharoenkul et al., 2024). The aforementioned variations in the prevalence, could be due to the differences in the diagnostic criteria used in the Asian and European regions (Petermann-Rocha et al., 2021). Besides that, tools used for the measurement of the appendicular skeletal mass influenced the reported prevalence. Dual-energy X-ray absorptiometry (DXA) has a better sensitivity and specificity compared to BIA (Cheng et al., 2021). Nonetheless, BIA was chosen as it was portable, lightweight, easily assembled and not time consuming (Kandayah et al., 2023). Moreover, studies have shown BIA as a suitable alternative to DXA and functions as an effective tool in diagnosing sarcopenia (Sousa-Santos et al., 2021; van den Helder et al., 2022).

Alarming high prevalence of ADL and IADL dependence, together with falls incidents in our study are consistent with the national elderly health survey findings in Malaysia (NIH, 2018b). This could be attributed to the fact that, majority of the respondents were from low income group, having primary education or less and are physically inactive. Studies globally have indicated that, these factors as significant predictors for functional status dependence and falls (Maruszewska, Ambroży & Rydzik, 2025; Hyejin et al., 2021; Nguyen et al., 2022).

### Risk factors of sarcopenia

Respondents in the age category of 75 years and above are one of the risk factors for severe sarcopenia. A similar finding was found in a study conducted in Thailand involving 330 respondents, which showed age as one of the risk factors for sarcopenia (Therakomen, Petchlorlian & Lakananurak, 2020). Loss of muscle mass accelerates significantly, especially after the age of 60 (Holloszy, 2000; Melton et al., 2000; Volpi, Nazemi & Fujita, 2004). Besides that, with increasing age, lower proportion of elderly participates in physical activity due to health conditions that restricts their mobility, fear of falling and depression (Meredith et al., 2023; Zhang & Jiang, 2023). In line with the concept of ‘use it or lose it’ in muscle metabolism, lack of physical activity, causes loss of muscle mass and strength which in turn increases the tendency for the elderly to suffer sarcopenia (Grgic, 2022).

We found that female gender as a risk factor for possible sarcopenia, confirmed sarcopenia and severe sarcopenia. According to a cohort study in United Kingdom, female gender had 20.0% higher risk of developing sarcopenia compared to male gender (Yang, Smith & Hamer, 2019). This could be due to the hormonal changes which causes skeletal muscle loss to occur more quickly in women than in men, especially from the age of 65 years to 74 years (Kodete et al., 2024; Burger et al., 2002). In contrast, a study in Brazil showed that elderly men had a higher risk of sarcopenia compared to the elderly women (Pelegrini et al., 2018). This could be due to the rapid reduction in testosterone and insulin-like growth factor-1 levels in elderly men which leads to loss of muscle mass and strength (Du et al., 2019).

Indian race is identified as a risk factor for severe sarcopenia. According to a study in done in India, Indian individuals were found to have high intramyocellular fat content in the skeletal muscle cells (Sucharita et al., 2019). High intramyocellular fat content were found to be detrimental to skeletal muscle density which subsequently increases the likelihood of developing sarcopenia (Rolland, 2008). Nonetheless, we cannot conclude based on this particular study alone that other races are at lower risk of developing sarcopenia as there are various factors apart from race, that influences the occurrence of sarcopenia (Cheng et al., 2021).

Based on the result, Petaling district which represents the urban area were identified as a risk factor for developing possible sarcopenia and severe sarcopenia which concurred with the result from a previous study in Unites States (Aziz et al., 2020). Although various recreational facilities are available in urban areas, these facilities are generally located far from the residential areas, making it difficult for the elderly to travel and exercise (Yu et al., 2019). Besides that, the food environment in urban area are saturated with unhealthy food options due to the increased availability and accessibility to fast food outlets (Kandayah, Safian & Shah, 2023). Subsequently, the likelihood of the elderly adopting unhealthy eating habits increases, which are proven to be harmful to overall muscle health (Zhang et al., 2020; van Erpecum et al., 2022).

Elderly having primary education or less were identified as a risk factor for severe sarcopenia. According to a study in Ireland which involved 3,342 respondents, elderly with low level of education often face socioeconomic disadvantages, making them more susceptible to sarcopenia compared to those with higher educational attainment (Swan, Warters & O’Sullivan, 2021). Low level of education often translates to poor health literacy which becomes a barrier to understand the importance of proper nutrition and exercise, particularly muscle resistance exercise which are essential to prevent sarcopenia (Fan et al., 2024).

In terms of physical activity, being physically inactive increases the risk for severe sarcopenia. Previous research finding have shown that being physically active helps to increase the muscle mass and strength and reduces the possibility of developing sarcopenia (Mijnarends et al., 2016). Another separate study done in China showed that, apart from increasing grip strength and muscle mass, exercise also improves physical performance of the elderly (Wang, Huang & Zhao, 2022). This underscores the importance of elderly being physical active which helps to reduce the risk of developing sarcopenia.

Respondents with type-2 diabetes mellitus are at risk of developing confirmed sarcopenia which are in line with a study conducted in primary care clinics in Malaysia (Sazlina et al., 2020). High intramyocellular fat in the skeletal muscle cell is linked to insulin resistance which is postulated to induce the muscle attenuation (Ahmed Al Saedi, Debruin & Hayes, 2022; Liu & Zhu, 2023). Besides that, various studies have indicated that diabetes mellitus could induce mitochondrial dysfunction which is proven as one of the key factor in the development of sarcopenia (Ferri et al., 2020; Chen et al., 2023).

Results showed that elderly with depression are susceptible for confirmed sarcopenia. Based on studies done in Taiwan and Turkey, elderly suffering from depression were found to be detached from social activities, resulting in physical inactivity and at a higher risk for poor dietary intake (Lin et al., 2024; Delibaş et al., 2021). Besides that, depression could be triggered by the sudden death of a spouse and increases the risk of institutionalization, particularly those with poor family support (Lu et al., 2023; Nihtilä & Martikainen, 2008). The aforementioned spillover effects, subsequently increases the tendency of the elderly developing sarcopenia.

Interestingly, we found that hypertension as a protective factor against confirmed sarcopenia which is not in line with various studies (Bai et al., 2020; Quan et al., 2023). However, there are several possible mechanism of how high blood pressure may serve as a protective factor for sarcopenia. A study done in Netherlands showed that elderly with high systolic, diastolic and mean arterial pressure (MAP), had a higher hand grip strength (Taekema et al., 2011). A separate study in Japan indicated that elderly with hypertension, together with a high CD 34-positive cell content to have a higher hand grip strength (Shimizu et al., 2021). High blood pressure causes injury to the endothelium resulting in platelet activation, which in turn causes an increase in CD 34-positive cells. The high content of CD 34-positive cells is postulated to play a role in stimulating angiogenesis and activating muscle satellite cells (Radu et al., 2023). However, it is important to take note that, further research with a bigger sample size is required to draw a conclusion as complex interaction between the diseases may lead to unexpected protective association of hypertension on sarcopenia.

### Effect of sarcopenia on functional status dependence

Confirmed sarcopenia and severe sarcopenia were identified as risk factors for ADL dependence. These findings align with a cohort study in Korea which reported 22.2% of respondents with sarcopenia had ADL dependence compared to only 10.9% of respondents without sarcopenia (Jang et al., 2018). Another study in Latin America and the Caribbean region indicated that decline in the gait speed as a mediating factor between sarcopenia and functional status dependence (Perez-Sousa et al., 2019).

Respondents with confirmed sarcopenia and severe sarcopenia were identified as risk factors for IADL dependence which is consistent with other previous study (Jang et al., 2018). Another separate study in Korea showed that, respondents with walking speed of less than 0.6 m/s were associated with IADL dependency (Hong et al., 2016). This could explain the findings in the current study, as gait speed which is a component of physical performance is impacted in possible sarcopenia, confirmed sarcopenia and severe sarcopenia (Chen et al., 2020).

### Falls characteristics and effect of sarcopenia

We have reported that most of the falls incidents occurred among individuals with possible sarcopenia, confirmed sarcopenia and severe sarcopenia. This further reinforces previous research findings that identified sarcopenia as a risk factor for falls, as muscle strength and muscle mass are crucial for maintaining a steady gait while walking (Yeung et al., 2019; Iskandar et al., 2021; Dhillon & Hasni, 2017). In terms of frequency of falls, most of the respondents who had any or recurrent falls, were those with possible sarcopenia, confirmed sarcopenia and severe sarcopenia. Studies have shown that sarcopenia affects the gait speed, endurance and balance which increases the tendency of recurrent falling (Iijima & Aoyama, 2021; Landi et al., 2012).

Majority of minor and severe injuries resulting from falls were experienced by respondents with possible sarcopenia, confirmed sarcopenia, or severe sarcopenia. A study in Korea showed that, elderly with sarcopenia had 1.6 times higher odds to suffer from falls-related injuries compared to those without sarcopenia (Woo & Kim, 2014). Slow gait speed which is observed in individuals with sarcopenia, causes severe injuries such as fractures during a fall event (Harris et al., 2022). Previous studies have indicated that, slow gait speed is associated with reduced bone mineral density in elderly (Kwon et al., 2007; Hirase et al., 2023). Besides that, elderly with slow gait speed have longer reaction time which directly impacts the severity of the injury sustained as the individual ability to react and adapt to the loss of balance during a fall event is compromised (Liu et al., 2024). Hence it was not surprising, as the majority of admission to the ward due to severe injuries in this study are among those elderly with possible sarcopenia, confirmed sarcopenia and severe sarcopenia.

Results shows that, most of the falls occurred inside the house compared to outside the house and majority of the respondents are those suffering from sarcopenia, irrespective of the classification. A cross-sectional study in Brazil showed that, extrinsic factors such as stairs, loose carpets, high toilet position and slippery floor inside the house may contribute as a hazard during a fall event (Rossetin et al., 2016). Apart from the extrinsic factors, the elderly may also suffer from vision impairment, which further escalates the risk of fall inside the house (Metanmo et al., 2022; Li et al., 2023). In addition, sarcopenia is also identified as a prominent risk factor for postural instability that increases the likelihood of falling (Kim et al., 2020). Based on a study in Japan, sarcopenia causes detrimental effects on the lower limb proprioception, which directly impacts the postural stability and subsequently increasing the risk of fall (Sakai et al., 2022).

### Limitation

This is a cross sectional study, hence we are unable to determine causal relationship. A larger sample size is required to further reduce the impact of confounding factors and gender representation. Besides that, this study used instruments such as the stop watch, hand grip dynamometer and BIA that increases the tendency of measurement bias. In order to address the issue, the instruments were calibrated accordingly. The researcher underwent training and certification with the Malaysian Society of Body Composition (MSBC) prior to the data collection. Apart from that, elderly at the care centre were not included which has limited the external validity of this study. The aforementioned limitations were inevitable due to hindrance in terms of time and resources.

## Conclusion

This study reveals concerning findings regarding the high prevalence of sarcopenia and its detrimental health effects on functional status dependence and falls incidence. Besides that, majority of the risk factors were modifiable in nature. Hence, it is important to initiate sarcopenia screening at the community level to identify the elderly at risk. Early identification will help the healthcare providers to prescribe interventions such as the muscle resistance exercise and nutritional interventions which are essential to mitigate the complications associated with sarcopenia.

## Supplemental Information

10.7717/peerj.20175/supp-1 Supplemental Information 1 Raw data.

10.7717/peerj.20175/supp-2 Supplemental Information 2 Codebook.

10.7717/peerj.20175/supp-3 Supplemental Information 3 STROBE Checklist.

## Additional Information and Declarations

### Field Study Permissions

The following information was supplied relating to field study approvals (*i.e*., approving body and any reference numbers):

Universiti Kebangsaan Malaysia.

### Data Availability

The following information was supplied regarding data availability:

The raw data is available in the Supplemental File.

## References

[ref-1] Ahmed Al Saedi MH, Debruin DA, Hayes A (2022). Lipid metabolism in sarcopenia. Bone.

[ref-2] Aziz J, Reid K, Batsis J, Fielding R (2020). Urban-rural differences in sarcopenia prevalence and nutritional risk factors: the NHANES (2001–2002 and 2011–2014). Innovation in Aging.

[ref-3] Bai T, Fang F, Li F, Ren Y, Hu J, Cao J (2020). Sarcopenia is associated with hypertension in older adults: a systematic review and meta-analysis. BMC Geriatrics.

[ref-4] Beaudart C, McCloskey E, Bruyère O, Cesari M, Rolland Y, Rizolli R, Carvalho IAD, Thiyagarajan JA, Bautmans I, Bertière MC, Brandi ML, Al-Daghri NM, Burlet N, Cavalier E, Cerreta F, Cherubini A, Fielding R, Gielin E, Landi F, Petermans J, Reginster JY, Visser M, Kanis J, Cooper C (2016). Sarcopenia in daily practice: assessment and management. BMC Geriatrics.

[ref-5] Burger HG, Dudley EC, Robertson DM, Dennerstein L (2002). Hormonal changes in the menopause transition. Recent Progress in Hormone Research.

[ref-6] Chen H, Huang X, Dong M, Wen S, Zhou L, Yuan X (2023). The association between sarcopenia and diabetes: from pathophysiology mechanism to therapeutic strategy. Diabetes, Metabolic Syndrome and Obesity.

[ref-7] Chen LK, Liu LK, Woo J, Assantachai P, Auyeung TW, Bahyah KS, Chou MY, Chen LY, Hsu PS, Krairit O, Lee JSW, Lee WJ, Lee Y, Liang CK, Limpawattana P, Lin CS, Peng LN, Satake S, Suzuki T, Won CW, Wu CH, Wu SN, Zhang T, Zeng P, Akishita M, Arai H (2014). Sarcopenia in Asia: consensus report of the Asian working group for Sarcopenia. Journal of the American Medical Directors Association.

[ref-8] Chen LK, Woo J, Assantachai P, Auyeung TW, Chou MY, Iijima K, Jang HC, Kang L, Kim M, Kim S, Kojima T, Kuzuya M, Lee JSW, Lee SY, Lee WJ, Lee Y, Liang CK, Lim JY, Lim WS, Peng LN, Sugimoto K, Tanaka T, Won CW, Yamada M, Zhang T, Akishita M, Arai H (2020). Asian working group for sarcopenia: 2019 consensus update on sarcopenia diagnosis and treatment. Journal of the American Medical Directors Association.

[ref-9] Cheng KY‐K, Chow SK‐H, Hung VW‐Y, Wong CH‐W, Wong RM‐Y, Tsang CS‐L, Kwok T, Cheung W‐H (2021). Diagnosis of sarcopenia by evaluating skeletal muscle mass by adjusted bioimpedance analysis validated with dual-energy X-ray absorptiometry. Journal of Cachexia, Sarcopenia and Muscle.

[ref-10] Cheng L, Sit JWH, Chan HYL, Choi KC, Cheung RKY, Wong MMH, Li FYK, Lee TY, Fung ESM, Tai KM, So WKW (2021). Sarcopenia risk and associated factors among Chinese community-dwelling older adults living alone. Scientific Reports.

[ref-11] Cruz-Jentoft AJ (2010). Sarcopenia: European consensus on definition and diagnosis report of the European Working Group on Sarcopenia in older people. Age and Ageing.

[ref-12] Cruz-Jentoft AJ, Bahat G, Bauer J, Boirie Y, Bruyère O, Cederholm T, Cooper C, Landi F, Rolland Y, Sayer AA, Schneider SM, Sieber CC, Topinkova E, Vandewoude M, Visser M, Zamboni M (2019). Sarcopenia: revised European consensus on definition and diagnosis. Age Ageing.

[ref-13] Delibaş DH, Eskut N, İlhan B, Erdogan E, Karti DT, Kusbeci OY, Bahat G (2021). Clarifying the relationship between sarcopenia and depression in geriatric outpatients. The Aging Male.

[ref-14] Dhillon RJS, Hasni S (2017). Pathogenesis and management of sarcopenia. Clinics in Geriatric Medicine.

[ref-15] DOSM (2021). Poket stats Negeri Selangor ST1 2021. https://library.dosm.gov.my/cgi-bin/koha/opac-detail.pl?biblionumber=104385.

[ref-16] DOSM (2024a). 11 daerah dikenal pasti sebagai daerah menua—DOSM, *Bernama*. https://www.astroawani.com/berita-malaysia/11-daerah-dikenal-pasti-sebagai-daerah-menua-dosm-485786.

[ref-17] DOSM (2024b). Current population estimates 2024. https://storage.dosm.gov.my/demography/population_2024.pdf.

[ref-18] Du Y, Wang X, Xie H, Zheng S, Wu X, Zhu X, Zhang X, Xue S, Li H, Hong W, Tang W, Chen M, Cheng Q, Sun J (2019). Sex differences in the prevalence and adverse outcomes of sarcopenia and sarcopenic obesity in community dwelling elderly in East China using the AWGS criteria. BMC Endocrine Disorders.

[ref-19] Fan H, Li M, Zhang C, Sun H, Shi S, Ma B (2024). Knowledge, attitude, and practice toward sarcopenia among older adults in two cities in Zhejiang province, China. Preventive Medicine Reports.

[ref-20] Ferri E, Marzetti E, Calvani R, Picca A, Cesari M, Arosio B (2020). Role of age-related mitochondrial dysfunction in sarcopenia. International Journal of Molecular Sciences.

[ref-21] Grgic J (2022). Use it or lose it? A meta-analysis on the effects of resistance training cessation (Detraining) on muscle size in older adults. International Journal of Environmental Research and Public Health.

[ref-22] Guerra-Farfan E, Garcia-Sanchez Y, Jornet-Gibert M, Nuñez JH, Balaguer-Castro M, Madden K (2023). Clinical practice guidelines: the good, the bad, and the ugly. Injury.

[ref-23] Harris JACRJ, Parimi N, Cawthon PM, Strotmeyer ES, Robert M, Boudreau JSB, Kwoh CK (2022). Associations of components of sarcopenia with risk of fracture in the osteoporotic fractures in men (MrOS) study rebekah. Osteoporosis International.

[ref-24] Hirase T, Okubo Y, Delbaere K, Menant JC, Lord SR, Sturnieks DL (2023). Risk factors for falls and fall-related fractures in community-living older people with pain: a prospective cohort study. International Journal of Environmental Research and Public Health.

[ref-25] Holloszy JO (2000). The biology of aging. Mayo Clinic Proceedings.

[ref-26] Hong S, Kim S, Yoo J, Kim BS, Choi HR, Choi SE, Hong CG, Won CW (2016). Slower gait speed predicts decline in instrumental activities of daily living in community-dwelling elderly: 3-year prospective finding from living profiles of older people survey in Korea. Journal of Clinical Gerontology and Geriatrics.

[ref-27] Hunter GR, Singh H, Carter SJ, Bryan DR, Fisher G (2019). Sarcopenia and its implications for metabolic health. Journal of Obesity.

[ref-28] Hyejin L, Bumjo O, Sunyoung K, Kiheon L (2021). ADL/IADL dependencies and unmet healthcare needs in older persons: a nationwide survey. Archives of Gerontology and Geriatrics.

[ref-29] Iijima H, Aoyama T (2021). Increased recurrent falls experience in older adults with coexisting of sarcopenia and knee osteoarthritis: a cross-sectional study. BMC Geriatrics.

[ref-30] Inbody (2016). PUSH-manual.pdf. https://inbodycanada.ca/wp-content/uploads/2020/06/PUSH-Manual.pdf.

[ref-31] Iskandar I, Joanny A, Azizan A, Justine M (2021). The prevalence of sarcopenia and its impact on quality of life in elderly residing in the community. Malaysian Journal of Medical Sciences.

[ref-32] Jang IY, Jung HW, Lee CK, Yu SS, Lee YS, Lee E (2018). Comparisons of predictive values of sarcopenia with different muscle mass indices in Korean rural older adults: a longitudinal analysis of the aging study of PyeongChang rural area. Clinical Interventions in Aging.

[ref-33] Jang JY, Kim D, Kim ND (2023). Pathogenesis, intervention, and current status of drug development for sarcopenia: a review. Biomedicines.

[ref-34] Kandayah T, Safian N, Shah SA (2023). Environmental factors associated with sarcopenia : a systematic review. International Journal of Public Health Research.

[ref-35] Kandayah T, Safian N, Shah SA, Manaf MRA (2023). Challenges in the management of sarcopenia in the primary care setting: a scoping review. International Journal of Environmental Research and Public Health.

[ref-36] Kim AY, Lee JK, Kim SH, Choi J, Song JJ, Chae SW (2020). Is postural dysfunction related to sarcopenia? A population-based study. PLOS ONE.

[ref-37] Kirk C-JA, Cawthon PM, Arai H, Ávila-Funes JA, Barazzoni R, Bhasin S, Binder EF, Bruyere O, Cederholm T, Chen LK, Cooper C, Duque G, Fielding RA, Guralnik J, Kiel DP, Landi F, Reginster JY, Sayer AA, Visser M, von Haehling S, Woo J (2024). Conceptual definition of sarcopenia—Delphi consensus from the global leadership initiative in sarcopenia. Chinese Journal of Geriatrics.

[ref-38] Kodete CS, Thuraka B, Pasupuleti V, Malisetty S (2024). Hormonal influences on skeletal muscle function in women across life stages: a systematic review. Muscles.

[ref-39] Kwon J, Suzuki T, Yoshida H, Kim H, Yoshida Y, Iwasa H, Sugiura M, Furuna T (2007). Association between change in bone mineral density and decline in usual walking speed in elderly community-dwelling Japanese women during 2 years of follow-up. Journal of the American Geriatrics Society.

[ref-40] Landi F, Liperoti R, Russo A, Giovannini S, Tosato M, Capoluongo E, Bernabei R, Onder G (2012). Sarcopenia as a risk factor for falls in elderly individuals: results from the ilSIRENTE study. Clinical Nutrition.

[ref-41] Lawton MP, Brody EM (1969). Assessment of older people: self-maintaining and instrumental activities of daily living. The Gerontologist.

[ref-42] Li Y, Hou L, Zhao H, Xie R, Yi Y, Ding X (2023). Risk factors for falls among community-dwelling older adults: a systematic review and meta-analysis. Frontiers of Medicine.

[ref-43] Lin YH, Han DS, Lee YH, Chan DC, Chang CH, Yang KC, Chang FC (2024). Social network associated with depressed mood and sarcopenia among older adults in Taiwan. Journal of the Formosan Medical Association.

[ref-44] Liu F, Yu H, Xu Q, Gong J, Huo M, Huang F (2024). Risk assessment of falls among older adults based on probe reaction time during water-carrying walking. Clinical Interventions in Aging.

[ref-45] Liu ZJ, Zhu CF (2023). Causal relationship between insulin resistance and sarcopenia. Diabetology & Metabolic Syndrome.

[ref-46] Lu L, Mao L, Yang S, He X, Zhang Z, Chen N (2023). Gender differences in the association between sarcopenia and depressive symptoms among community-dwelling older people in a Chinese Suburban Area. Journal of Multidisciplinary Healthcare.

[ref-47] Mahoney DW, Barthel FI (1965). Functional evaluation: the barthel index. Maryland State Medical Journal.

[ref-48] Maruszewska A, Ambroży T, Rydzik Ł (2025). Risk factors and socioeconomic determinants of falls among older adults. Frontiers in Public Health.

[ref-49] Melton BLRLJ, Khosla S, Cynthia S, Crowson BS, O’Connor MK, O’Fallon WM (2000). Epidemiology of Sarcopenia. Journal of the American Geriatrics Society.

[ref-50] Meredith SJ, Cox NJ, Ibrahim K, Higson J, McNiff J, Mitchell S, Rutherford M, Wijayendran A, Shenkin SD, Kilgour AHM, Lim SER (2023). Factors that influence older adults’ participation in physical activity: a systematic review of qualitative studies. Age and Ageing.

[ref-51] Metanmo S, Tegueu CK, Gbessemehlan A, Dartigues JF, Ntsama MJ, Yonta LN, Kengne AP, Tabue NS, Teguo MT (2022). Self-reported visual impairment and sarcopenia among older people in Cameroon. Scientific Reports.

[ref-52] Mijnarends DM, Koster A, Schols JMGA, Meijers JMM, Halfens RDG, Gudnason V, Eiriksdottir G, Siggeirsdottir K, Sigurdsson S, Jonsson PV, Meirelles O, Harris T (2016). Physical activity and incidence of sarcopenia: the population-based AGES-Reykjavik study. Age and Ageing.

[ref-53] Mohamad Zani RA, Ahmad Yusof H, Ain Azizan N, Hyder Ali IA, Ismail S, Mohd Shariff N (2025). Sarcopenia and it’s influencing factors among adults with asthma, chronic obstructive pulmonary disease, and tuberculosis in Penang, Malaysia. BMC Public Health.

[ref-54] Moreira-Pais A, Ferreira R, Oliveira PA, Duarte JA (2022). A neuromuscular perspective of sarcopenia pathogenesis: deciphering the signaling pathways involved. GeroScience.

[ref-55] Murph CH, McCarthy SN, McMorrow AM, Egan B, McGowan MJ, Rafferty S, Corish CA, Roche HM (2023). Prevalence and determinants of sarcopenia in community-dwelling older adults in Ireland. Aging Clinical and Experimental Research.

[ref-56] Nemeth R (2002). Respondent selection within the household—a modification of the Kish grid 1 introduction 2 reasons for sampling households 3 the Kish grid.

[ref-57] Nguyen VC, Moon SH, Oh E, Hong GRS (2022). Factors associated with functional limitations in daily living among older adults in korea: a cross-sectional study. International Journal of Public Health.

[ref-58] NIH (2018a). National health and morbidity survey 2018: elderly health volume one: methodology and general findings. https://iku.moh.gov.my/images/IKU/Document/REPORT/NHMS2018/NHMS2018ElderlyHealthVolume1.pdf.

[ref-59] NIH (2018b). National health and morbidity survey 2018: elderly health. volume two: elderly health findings. https://iku.moh.gov.my/images/IKU/Document/REPORT/NHMS2018/NHMS2018ElderlyHealthVolume2.pdf.

[ref-60] Nihtilä E, Martikainen P (2008). Institutionalization of older adults after the death of a spouse. American Journal of Public Health.

[ref-61] Pang BWJ, Wee SL, Lau LK, Jabbar KA, Seah WT, Ng DHM, Tan QLL, Chen KK, Jagadish MU, Ng TP (2021). Prevalence and associated factors of sarcopenia in singaporean adults—the yishun study. Journal of the American Medical Directors Association.

[ref-62] Patel HP, White MC, Westbury L, Syddall HE, Stephens PJ, Clough GF, Cooper C, Sayer AA (2015). Skeletal muscle morphology in sarcopenia defined using the EWGSOP criteria: findings from the Hertfordshire Sarcopenia Study (HSS) Physical functioning, physical health and activity. BMC Geriatrics.

[ref-63] Pelegrini A, Mazo GZ, de Pinto AA, Benedetti TRB, Silva DAS, Petroski EL (2018). Sarcopenia: prevalence and associated factors among elderly from a Brazilian capital. Fisioterapia em Movimento.

[ref-64] Perez-Sousa MA, Sanabria LCV, Carvajal DAC, Gutierrez CAC, Izquierdo M, Bautista JEC, Velez RR (2019). Gait speed as a mediator of the effect of sarcopenia on dependency in activities of daily living. Journal of Cachexia, Sarcopenia and Muscle.

[ref-65] Petermann-Rocha F, Balntzi V, Gray SR, Lara J, Ho FK, Jill P, Pell JP, Celis-Morales C (2021). Global prevalence of sarcopenia and severe sarcopenia: a systematic review and meta-analysis. Journal of Cachexia, Sarcopenia and Muscle.

[ref-66] Quan Y, Wang C, Wang L, Li G (2023). Geriatric sarcopenia is associated with hypertension: a systematic review and meta-analysis. Journal of Clinical Hypertension.

[ref-67] Radu P, Zurzu M, Paic V, Bartucu M, Garofil D, Tigora A, Georgescu V, Prunoiu V, Pasnicu C, Popa F, Surlin P, Surlin V, Strambu V (2023). CD34—structure, functions and relationship with cancer stem cells. Medicina.

[ref-68] Ramoo K, Hairi NN, Yahya A, Choo WY, Hairi FM, Peramalah D, Kandiben S, Bulgiba A, Ali ZM, Razak IA, Ismail N, Ahmad NS (2022). Longitudinal association between sarcopenia and cognitive impairment among older adults in rural Malaysia. International Journal of Environmental Research and Public Health.

[ref-69] Ranee R, Shahar S, You YX, Singh DKA, Sakian NIM (2022). Prevalence and risk factors of sarcopenia among community dwelling older adults in Klang Valley. Malaysian Journal of Medicine and Health Sciences.

[ref-70] Rolland Y (2008). Sarcopenia: its assessment, etiology, pathogenesis, consequences and future perspectives. The Journal of Nutrition, Health and Aging.

[ref-71] Rosenberg IH (1989). Summary comments. American Journal of Clinical Nutrition.

[ref-72] Rosenberg IH (1997). Sarcopenia: origins and clinical relevance. The Journal of Nutrition.

[ref-73] Rossetin LL, Rodrigues EV, Gallo LH, Macedo DS, Schieferdecker MEM, Pintarelli VL, Rabito EI, Gomes ARS (2016). Indicadores de sarcopenia e sua relação com fatores intrínsecos e extrínsecos às quedas em idosas ativas. Revista Brasileira de Geriatria e Gerontologia.

[ref-74] Sakai Y, Watanabe T, Wakao N, Matsui H, Osada N, Sugiura T, Morita Y, Kawai K, Ito T, Yamazaki K (2022). Proprioception and geriatric low back pain. Spine Surgery and Related Research.

[ref-75] Sazlina S-G, Lee PY, Chan YM, Hamid MSA, Tan NC (2020). The prevalence and factors associated with sarcopenia among community living elderly with type 2 diabetes mellitus in primary care clinics in Malaysia. PLOS ONE.

[ref-76] Shimizu Y, Kawashiri SY, Nobusue K, Yamanashi H, Nagata Y, Maeda T (2021). Associations between handgrip strength and hypertension in relation to circulating CD34-positive cell levels among Japanese older men: a cross-sectional study. Environmental Health and Preventive Medicine.

[ref-77] Sousa-Santos AR, Barros D, Montanha TL, Carvalho J, Amaral TF (2021). Which is the best alternative to estimate muscle mass for sarcopenia diagnosis when DXA is unavailable?. Archives of Gerontology and Geriatrics.

[ref-78] Sucharita S, Pranathi R, Correa M, Keerthana P, Ramesh LJ, Bantwal G, Venkatappa HM, Mahadev KP, Thomas T, Bosch RJ, Harridge SDR, Kurpad AV (2019). Evidence of higher intramyocellular fat among normal and overweight Indians with prediabetes. European Journal of Clinical Nutrition.

[ref-79] Swan L, Warters A, O’Sullivan M (2021). Socioeconomic inequality and risk of sarcopenia in community-dwelling older adults. Clinical Interventions in Aging.

[ref-80] Taekema DG, Maier AB, Westendorp RGJ, De Craen AJM (2011). Higher blood pressure is associated with higher handgrip strength in the oldest old. American Journal of Hypertension.

[ref-81] Tarantino G, Sinatti G, Citro V, Santini SJ, Balsano C (2023). Sarcopenia, a condition shared by various diseases: can we alleviate or delay the progression?. Intern Emergency Medicine.

[ref-82] Therakomen V, Petchlorlian A, Lakananurak N (2020). Prevalence and risk factors of primary sarcopenia in community-dwelling outpatient elderly: a cross-sectional study. Scientific Reports.

[ref-83] van den Helder J, Verreijen AM, Dronkelaar CV, Memelink RG, Engberink MF, Engelbert RHH, Weijs PJM, Tieland M (2022). Bio-electrical impedance analysis: a valid assessment tool for diagnosis of low appendicular lean mass in older adults?. Frontiers in Nutrition.

[ref-84] van Erpecum CPL, van Zon SKR, Bültmann U, Smidt N (2022). The association between fast-food outlet proximity and density and body mass index: findings from 147,027 lifelines cohort study participants. Preventive Medicine.

[ref-85] Vanitcharoenkul E, Unnanuntana A, Chotiyarnwong P, Laohaprasitiporn P, Adulkasem N, Asavamongkolkul A, Chandhanayingyong C (2024). Sarcopenia in Thai community-dwelling older adults: a national, cross-sectional, epidemiological study of prevalence and risk factors. BMC Public Health.

[ref-86] Volpi E, Nazemi R, Fujita S (2004). Muscle tissue changes with aging. Current Opinion in Clinical Nutrition and Metabolic Care.

[ref-87] Wang H, Huang Y, Zhao Y (2022). Efficacy of Exercise on muscle function and physical performance in older adults with sarcopenia: an updated systematic review and meta-analysis. International Journal of Environmental Research and Public Health.

[ref-88] Wearing J, Konings P, De Bie RA, Stokes M, De Bruin ED (2020). Prevalence of probable sarcopenia in community-dwelling older Swiss people—a cross-sectional study. BMC Geriatrics.

[ref-89] WHO (2002). Global physical activity questionnaire (GPAQ): analysis guide. https://www.who.int/ncds/surveillance/steps/resources/GPAQ_Analysis_Guide.pdf.

[ref-90] Woo N, Kim SH (2014). Sarcopenia influences fall-related injuries in community-dwelling older adults. Geriatric Nursing.

[ref-91] Xie WQ, He M, Yu DJ, Wu YX, Wang XH, Lv S, Xiao WF, Li YS (2021). Mouse models of sarcopenia: classification and evaluation. Journal of Cachexia, Sarcopenia and Muscle.

[ref-92] Yang L, Smith L, Hamer M (2019). Gender-specific risk factors for incident sarcopenia: 8-year follow-up of the English longitudinal study of ageing. Journal of Epidemiology and Community Health.

[ref-93] Yesavage JA, Brink TL, Rose TL, Lum O, Huang V, Adey M, Leirer VO (1982). Leirer development and validation of a geriatric depression screening scale: a preliminary report. Journal of Psychiatric Research.

[ref-94] Yeung SSY, Reijnierse EM, Pham VK, Trappenburg MC, Lim WK, Meskers CGM, Maier AB (2019). Sarcopenia and its association with falls and fractures in older adults: a systematic review and meta-analysis. Journal of Cachexia, Sarcopenia and Muscle.

[ref-95] Yu S, Liu Y, Cui C, Xia B (2019). Influence of outdoor living environment on elders’ quality of life in old residential communities. Sustainability.

[ref-96] Yuan S, Larsson SC (2023). Epidemiology of sarcopenia: prevalence, risk factors, and consequences. Metabolism.

[ref-97] Zhang M, Guo W, Zhang N, He H, Zhang Y, Zhou M, Zhang J, Li M, Ma G (2020). Association between neighborhood food environment and body mass index among older adults in Beijing, China: a cross-sectional study. International Journal of Environmental Research and Public Health.

[ref-98] Zhang Y, Jiang X (2023). The effects of physical activity and exercise therapy on frail elderly depression: a narrative review. Medicine.

