# Supplementary material for: Prevalence and risk factors of sarcopenia and effect of sarcopenia on functional status and falls incidents among the elderly in Selangor
Source: PeerJ. 2025 Oct 17;13:e20175. doi: 10.7717/peerj.20175 (PMC12536799; doi:10.7717/peerj.20175)
Supplement: Supplemental Information 2 [file peerj-13-20175-s002.docx]

Codebook for multinomial logistic regression

With the use of SPSS, the choice of the reference category for multinomial logistic regression were coded by the highest category (by default) as the reference

| Variable | Values | Coding number | Reference category |
| --- | --- | --- | --- |
| Age | 60-74 years old | 0 |  |
|  | >75 years old | 1 | >75 years old |
|  |  |  |  |
| Gender | Female | 0 |  |
|  | Male | 1 | Male |
|  |  |  |  |
| Locality | Petaling | 0 |  |
|  | Kuala Langat | 1 | Kuala Langat |
|  |  |  |  |
| Race | Malay | 0 |  |
|  | Indian | 1 |  |
|  | Chinese | 2 | Chinese |
|  |  |  |  |
| Marital status | Single | 0 |  |
|  | Widowed or divorced | 1 |  |
|  | Married | 2 | Married |
|  |  |  |  |
| Level of education | Primary or less | 0 |  |
|  | Secondary | 1 |  |
|  | Tertiary | 2 | Tertiary |
|  |  |  |  |
| Occupation | Not employed | 0 |  |
|  | Housewife | 1 |  |
|  | Retired | 2 |  |
|  | Working | 3 | Working |
|  |  |  |  |
| Household income | B-40 (<RM 5250) | 0 |  |
|  | M-40 (RM 5250-RM11819) | 1 |  |
|  | T-20 (>RM 11820) | 2 | T-20 |
|  |  |  |  |
| Smoking status | Active smoker | 0 |  |
|  | Ex-smoker | 1 |  |
|  | Never smoked | 2 | Never smoked |
|  |  |  |  |
| Alcohol consumption status | Alcohol drinker | 0 |  |
|  | Ex-drinker | 1 |  |
|  | Never drinked | 2 | Never drinked |
|  |  |  |  |
| Physical activity | Inactive | 0 |  |
|  | Active | 1 | Active |
|  |  |  |  |
| DM-type 2 | Yes | 0 |  |
|  | No | 1 | No |
| Variable | Values | Coding number | Reference category |
| Hypertension | Yes | 0 |  |
|  | No | 1 | No |
|  |  |  |  |
| Dyslipidemia | Yes | 0 |  |
|  | No | 1 | No |
|  |  |  |  |
| Cardiovascular disease | Yes | 0 |  |
|  | No | 1 | No |
|  |  |  |  |
| Osteoporosis | Yes | 0 |  |
|  | No | 1 | No |
|  |  |  |  |
| Depression | Yes | 0 |  |
|  | No | 1 | No |

Codebook for binary logistic regression

For binary logistic regression, reference group(lowest risk) were coded by the lowest category.

| Variable | Values | Coding number | Reference category |
| --- | --- | --- | --- |
| Falls incidents | Yes | 1 |  |
|  | No | 0 | No |
|  |  |  |  |
| ADL dependency | Yes | 1 |  |
|  | No | 0 | No |
|  |  |  |  |
| IADL dependency | Yes | 1 |  |
|  | No | 0 | No |
|  |  |  |  |
| Sarcopenia classification | No sarcopenia | 0 | No sarcopenia |
|  | Possible sarcopenia | 1 |  |
|  | Sarcopenia and severe sarcopenia | 2 |  |
