# Supplementary material for: Prevalence and risk factors of sarcopenia and effect of sarcopenia on functional status and falls incidents among the elderly in Selangor
Source: PeerJ. 2025 Oct 17;13:e20175. doi: 10.7717/peerj.20175 (PMC12536799; doi:10.7717/peerj.20175)
Supplement: Supplemental Information 3 [file peerj-13-20175-s003.docx]

**Supplementary Table 1 :Strobe Statement Completed Checklist**

| **Section/Topic** | **Item No** | **STROBE Recommendation** | **Our paper** |
| --- | --- | --- | --- |
| **Title and abstract** | 1 | (a) Indicate the study’s design with a commonly used term in the title or the abstract | See title section |
|  |  | (b) Provide in the abstract an informative and balanced summary of what was done and what was found | See abstract section |
| **Introduction** |  |  |  |
| Background/rationale | 2 | Explain the scientific background and rationale for the investigation being reported | See Introduction section paragraph 4 which explains the rationale |
| Objectives | 3 | State specific objectives, including any prespecified hypotheses | See abstract section |
| **Methods** |  |  |  |
| Study design | 4 | Present key elements of study design early in the paper | See design and the study population section, paragraph 1 |
| Setting | 5 | Describe the setting, locations, and relevant dates, including periods of recruitment, exposure, follow-up, and data collection | See design and the study population section, paragraph 1 |
| Participants | 6 | Cross-sectional study—Give the eligibility criteria, and the sources and methods of selection of participants | See design and the study population section, paragraph 1 |
| Variables | 7 | Clearly define all outcomes, exposures, predictors, potential confounders, and effect modifiers. Give diagnostic criteria, if applicable | See study variables section, paragraph 1 |
| Data sources/ measurement | 8 | For each variable of interest, give sources of data and details of methods of assessment (measurement). Describe comparability of assessment methods if there is more than one group | a)See study variables section, paragraph 1  b) See diagnosis of sarcopenia section, paragraph 1 |
| Bias | 9 | Describe any efforts to address potential sources of bias | See limitation section, paragraph 1 |
| Study size | 10 | Explain how the study size was arrived at | See design and the study population section, paragraph 2 |
| Quantitative variables | 11 | Explain how quantitative variables were handled in the analyses. If applicable, describe which groupings were chosen and why | See data analysis section, paragraph 1 |
| Statistical methods | 12 | (a) Describe all statistical methods, including those used to control for confounding | See data analysis section, paragraph 1 |
|  |  | (b) Describe any methods used to examine subgroups and interactions | See data analysis section, paragraph 1 |
|  |  | (c) Explain how missing data were addressed | See data analysis section, paragraph 1 |
|  |  | (d)Cross-sectional study—If applicable, describe analytical methods taking account of sampling strategy | See design and the study population section, paragraph 1, explained on the multistage cluster sampling and usage of design effect of 1.2 |
|  |  | (e) Describe any sensitivity analyses | Not applicable |
|  |  |  |  |
| **Results** |  |  |  |
| Participants | 13 | (a) Report numbers of individuals at each stage of study—eg numbers potentially eligible, examined for eligibility, confirmed eligible, included in the study, completing follow-up, and analysed | See design and the study population section, paragraph 1 |
|  |  | (b) Give reasons for non-participation at each stage | See design and the study population section, paragraph 2 |
|  |  | (c) Consider use of a flow diagram | Not applicable |
| Descriptive data | 14 | (a) Give characteristics of study participants (eg demographic, clinical, social) and information on exposures and potential confounders | See results section, paragraph 1 |
|  |  | (b) Indicate number of participants with missing data for each variable of interest | Not applicable |
|  |  | (c) Cohort study—Summarise follow-up time (eg, average and total amount) | Not applicable |
| Outcome data | 15 | Cross-sectional study—Report numbers of outcome events or summary measures | See results section, paragraph 1 and paragraph 2.  See results section, Table 1 |
| Main results | 16 | (a) Give unadjusted estimates and, if applicable, confounder-adjusted estimates and their precision (eg, 95% confidence interval). Make clear which confounders were adjusted for and why they were included | See data analysis section, paragraph 1  See results section, Table (2,3,4,5) |
|  |  | (b) Report category boundaries when continuous variables were categorized | Not applicable |
|  |  | (c) If relevant, consider translating estimates of relative risk into absolute risk for a meaningful time period | Not applicable |
| Other analyses | 17 | Report other analyses done—eg analyses of subgroups and interactions, and sensitivity analyses | Not applicable |
| **Discussion** |  |  |  |
| Key results | 18 | Summarise key results with reference to study objectives | See discussion section , paragraph 1 and 2 |
| Limitations | 19 | Discuss limitations of the study, taking into account sources of potential bias or imprecision. Discuss both direction and magnitude of any potential bias | See limitation section, paragraph 1 |
| Interpretation | 20 | Give a cautious overall interpretation of results considering objectives, limitations, multiplicity of analyses, results from similar studies, and other relevant evidence | See conclusion section, paragraph 1 |
| Generalisability | 21 | Discuss the generalisability (external validity) of the study results | See limitation section, paragraph 1 |
| **Other information** |  |  |  |
| Funding | 22 | Give the source of funding and the role of the funders for the present study and, if applicable, for the original study on which the present article is based | See funding section. |

*Give information separately for cases and controls in case-control studies and, if applicable, for exposed and unexposed groups in cohort and cross-sectional studies.

Note: An Explanation and Elaboration article discusses each checklist item and gives methodological background and published examples of transparent reporting. The STROBE checklist is best used in conjunction with this article (freely available on the Web sites of PLoS Medicine at http://www.plosmedicine.org/, Annals of Internal Medicine at http://www.annals.org/, and Epidemiology at http://www.epidem.com/). Information on the STROBE Initiative is available at www.strobe-statement.org.
